# Measuring linkage to HIV treatment services following HIV self‐testing in low‐income settings

**DOI:** 10.1002/jia2.25548

**Published:** 2020-06-24

**Authors:** Augustine T Choko, Muhammad S Jamil, Peter MacPherson, Elizabeth Corbett, Lastone Chitembo, Heather Ingold, Elkin Bermudez Aza, Marc d’Elbee, Meghan DiCarlo, Mohammed Majam, Tanya Schewchuk, Vincent Wong, Rachel Baggaley, Cheryl Johnson, the World Health Organization HIV self‐testing technical working group

**Affiliations:** ^1^ TB/HIV Group Malawi Liverpool Wellcome Trust Clinical Research Programme Blantyre Malawi; ^2^ Department of Infectious Disease Epidemiology London School of Hygiene and Tropical Medicine London United Kingdom; ^3^ Global HIV, Hepatitis and SITs Programmes World Health Organization Geneva Switzerland; ^4^ Department of Clinical Sciences Liverpool School of Tropical Medicine Liverpool United Kingdom; ^5^ Department of Clinical Research London School of Hygiene and Tropical Medicine London United Kingdom; ^6^ World Health Organization Country Office for Zambia Lusaka Zambia; ^7^ Unitaid Geneva Switzerland; ^8^ Médecins Sans Frontières Holland Amsterdam Netherlands; ^9^ Department of Global Health and Development London School of Hygiene and Tropical Medicine London United Kingdom; ^10^ FHI 360 Washington DC USA; ^11^ Wits Reproductive Health and HIV Research Institute Johannesburg South Africa; ^12^ Bill and Melinda Gates Foundation Seattle WA USA; ^13^ Global Health Bureau Office of HIV/AIDS, Prevention Care and Treatment Division United States Agency for International Development (USAID) Arlington VA USA

**Keywords:** HIV testing services, HIV self‐testing, linkage, HIV care, monitoring and evaluation

Globally, HIV testing services (HTS) have been scaled up resulting in 79% of all people with HIV aware of their status in 2018 [1]. However, 8.1 million people remain undiagnosed [1], many of whom are hard to reach through traditional HTS approaches. In 2016, the World Health Organization (WHO) strongly recommended HIV self‐testing (HIVST) as an HTS approach, followed by an update in 2019 [2, 3]. Since 2016, the number of countries with supportive HIVST policies has grown rapidly to 77 with 38 countries implementing HIVST as of July 2019 [1]. HIVST has proved effective in reaching people with undiagnosed HIV and those at high ongoing risk [4, 5, 6], however, many countries are yet to implement or scale up HIVST.

As with any HTS, HIVST needs to provide a pathway to appropriate HIV treatment, care and prevention services. Because no single test, including HIVST, can provide an HIV‐positive diagnosis, all individuals with reactive HIVST results must receive further testing by a trained provider before initiating antiretroviral therapy (ART) [5]. Measuring linkage to ART is important to demonstrate programme effectiveness and impact, however, monitoring linkage after HIVST can be challenging because of its private nature. We highlight key challenges in measuring linkage to treatment and care following HIVST and suggest pragmatic approaches to addressing these in low‐income settings that routinely offer HIVST.

Evidence from randomized trials shows that the proportion of people linked to ART following HIVST is comparable to that of standard facility‐based HTS [5]. However, outside a research or trial environment, it may be unclear whether routine programmatic HIVST implementation results in similar successes. The challenges to accurately measuring linkage following HIVST include: (i) not knowing the number of HIVST kits used out of the number distributed, particularly when distributed in the community or via secondary distribution to partners and/or social contacts; (ii) clients on ART using HIVST to “check” their HIV status without disclosing their HIV‐positive status and/or ART use to the provider (kit distributor); (iii) clients using HIVST as a prompt for “re‐engaging” in care or “restarting” ART without disclosing their HIV‐positive status and/or ART use to the provider (kit distributor) [7]; (iv) clients with reactive HIVST results who are lost to follow‐up; and (v) use of paper‐based and unlinked clinic records, such as clinic registers or logbooks and the lack of case‐based surveillance in many low‐income settings, leading to duplicate or missing information, an issue affecting HTS monitoring broadly.

Given these challenges, measuring linkage in programmes at the individual level following HIVST may not be feasible in many low‐income settings. Such an effort may require repeated follow‐up with self‐testers which can be resource intensive in the absence of client information and linked electronic records. Attempts by providers to ascertain the client’s HIVST results through repeated follow‐up can also be perceived as being against client autonomy and could deter HIVST utilization in the future. Concerns about monitoring linkage have kept several national HIV programmes from making HIVST available to clients. We believe that resource intensive monitoring efforts should not come in the way of making HIVST widely available at the earliest. We give below a few pragmatic approaches to measuring linkage to treatment services that programmes can consider and adapt depending on their local context.

- *Monitor ART initiations at treatment centres/facilities before and during HIVST distribution in the relevant catchment area* [8, 9]. For example use of this approach in the HIV Self‐Testing Africa (STAR) Initiative showed a significant 27% increase in ART initiations after introduction of HIVST [9]. This approach will require the availability of reliable ART programme data and will be most useful for settings rapidly scaling HIVST and seeking to achieve high coverage in specific geographical settings and/or focus populations. These data will give an indication of whether target populations are being reached by HIVST kit distribution and whether those who are diagnosed are effectively linked to ART. However, careful interpretation is needed as increases in ART initiations may not be attributable to HIVST alone, and can be due to broader efforts to expand testing and demand creation initiatives within the catchment area.
- *Include questions in clinic registers that can help to ascertain if the present clinic visit/testing was prompted by prior HIVST use*. It is important to note that these data are subject to recall bias and some people may not disclose prior HIVST use and/or results for reasons such as stigma or to get a result from the provider without biases. These data also do not provide a denominator to measure linkage following HIVST. Nonetheless, such data can provide useful information on the proportion of ART initiations prompted by HIVST.
- *Population‐based surveys such as demographic and health surveys, integrated bio‐behavioural surveys and other special surveys provide opportunities to monitor HIVST use and linkage at the population level*. These surveys are particularly useful for monitoring trends over time, such as awareness, use, coverage and linkage, provided appropriate questions are included. Because these surveys are usually repeated every three to five years, they may not be useful for ongoing programme monitoring.
- *Digital tools, such as messaging Apps, websites, hotlines and social media platforms, can also be leveraged to collect HIVST usage and linkage information*. For example in South Africa, a survey of a random sample of self‐testers through mHealth platforms such as interactive voice response and SMS was found feasible for estimating HIVST usage and linkage [10].
- Lastly, *individual‐level follow‐up* to confirm linkage may be considered in the context of small‐scale demonstration projects or within research studies to assess the effectiveness of linkage interventions. One example of such an approach is when women in antenatal care distribute HIVST kits to their partners. Women can be given an invitation letter for their partner along with a self‐test kit. The male partners are asked to show the invitation letter when they attend clinics for HTS and women can also be interviewed at their second visit [11]. The linkage among male partners after HIVST may be an underestimate if they do not present the letter, seek testing at another clinic or already know their HIV‐status or are on ART. Individual‐level follow‐up to assess linkage will not be feasible in resource‐limited settings as programmes scale up due to the extensive resources required.

No single method would give an accurate measure of linkage following HIVST due to the limitations of each of them. However, using data and information from diverse sources, such as survey and programme data, can increase confidence in linkage estimates and minimize missing information. WHO is developing guidance for countries to monitor and evaluate HIVST, including linkage.

HIVST is an important testing approach for meeting the global goals of diagnosing 95% of all people with HIV by 2025. Effective linkage to appropriate services following HIVST is important. Given the privacy of HIVST, which allows autonomy, fosters empowerment and reaches people who may not otherwise test, a resource‐intensive approach to monitor linkage is neither feasible nor desirable as programmes scale up HIVST. The need to collect in‐depth linkage data should not delay the wider availability of HIVST. Programmes, donors and implementers should consider pragmatic and innovative ways to measure linkage.

## COMPETING INTERESTS

The authors declare no conflicts of interest.

## AUTHORS’ CONTRIBUTIONS

MSJ, CJ, ATC and PM conceived the idea. ATC wrote the first draft of manuscript. MSJ, PM, EC, EC, LC, HI, EBA, MdE, MDC, MM, TS, VW, RB and CJ reviewed, provided input and approved the final draft of manuscript.

## FUNDING

Bill and Melinda Gates Foundation OPP1177903 and Unitaid (PO# 10140–0‐600 and PO# 8477–0‐600). PM is funded by the Wellcome Trust (206575/Z/17/Z). EC is funded under a Wellcome Trust Senior Research Fellowship in Clinical Science (grant number: WT091769) and by Unitaid‐STAR Initiative (NCT02718274).

### DISCLAIMER

The authors alone are responsible for the views expressed in this article and they do not necessarily represent the views, decisions or policies of the institutions with which they are affiliated including the World Health Organization, the U.S. President's Emergency Plan for AIDS Relief, the U.S. Agency for International Development and the U.S. Government. The corresponding author had final responsibility for the decision to submit for publication.
